# Experiences of Humanizing Care in Nursing Students—A Phenomenological Study

**DOI:** 10.3390/healthcare13202569

**Published:** 2025-10-13

**Authors:** María Fernanda Valle Dávila, Cristina Fernanda Vaca Orellana, Silvia Lorena Acosta Balseca, Yrene Esperanza Urbina Rojas

**Affiliations:** 1 Facultad Ciencias de la Salud, Universidad Técnica del Norte, Ibarra 100150, Ecuador; 2 Facultad Ciencias de la Salud, Universidad Nacional de Tumbes, Tumbes 24001, Peru

**Keywords:** students, nursing, humanization of assistance, nurse-patient relations, qualitative research, education, professional competence

## Abstract

**Background:** Human care represents the essence of nursing but faces challenges from increasing technological advancement and healthcare system bureaucratization. **Objective:** To understand how nursing students balance technical demands with human aspects of care during pre-professional practice experiences. **Methods:** An interpretive phenomenological study was conducted with 17 nursing students (12 women, 5 men) in their eighth and ninth semesters from a public university in northern Ecuador. The data were collected through focused interviews during the first quarter of 2025. Analysis followed a four-stage phenomenological process: epoché, phenomenological reduction, eidetic reduction, and transcendental reduction, culminating in phenomenological interpretation. Data saturation was achieved, and methodological rigor criteria were applied including triangulation with external analysts. **Results:** Six main strategies emerged that students develop to balance technical demands with humanized care: Time Management and Optimization, Integration of Human and Technical Dimensions, Patient Communication About Time Constraints, Emotional Regulation and Boundary Setting, Resistance to Dehumanization, and Institutional Context Adaptation. Students transform technical procedures into therapeutic opportunities and develop resilient competencies that preserve nursing’s humanistic values. **Conclusions:** Nursing students develop integrative competencies that balance technical excellence with human sensitivity. Curriculum modifications are needed to include specific competencies in emotional regulation, therapeutic communication, and dehumanization resistance strategies.

## 1. Introduction

### 1.1. The Concept of Humanization in Care and International Theoretical Framework

Nursing practice constitutes both discipline and professional endeavor centered on therapeutic relationships. Yet contemporary healthcare environments face growing technological advancement, bureaucratization, and efficiency pressures that challenge care’s humanistic dimension and threaten to reduce it to technical procedures [1,2,3]. Training future nursing professionals thus becomes crucial. During this formative stage, students develop technical competencies while learning to understand, experience, and embody caring as fundamentally therapeutic practice [4,5,6].

Humanized care encompasses communication, mutual support, understanding, and compassion as essential elements tied to nursing’s foundational principles, values, and social, technical, scientific, ethical, and human dimensions. The World Health Organization [7] emphasizes comprehensive nursing professional training based on humanized care, requiring supervision to ensure quality care provision standards.

The documented tension between technical demands and humanistic value preservation has been identified as a conflict source in nursing students’ professional practice adaptation [8,9,10]. Duchscher [11] describes a transition process focusing on roles, responsibilities, relationships, knowledge, and humanistic ideals internalized during theoretical training as graduates experience professional environments.

While nursing education currently incorporates various theories guiding daily practice, along with institutional standards, practice guidelines, diagnoses, interventions, and outcomes based on established taxonomies, we must reflect on whether this knowledge wealth aligns with care ontology and whether academic training truly enables students to experience authentic encounters with those they serve [12].

### 1.2. The Latin American and Ecuadorian Context

Previous research on nursing student experiences includes qualitative and quantitative studies examining various aspects of this phenomenon: professional identity development [13], care meanings [14], clinical competency acquisition [15], and education-to-practice transition challenges [16]. However, limited phenomenological research specifically explores how students experience environmental dehumanizing pressures, particularly within Latin American contexts where specific sociocultural, economic, and institutional factors significantly influence these experiences.

In Ecuador, professional training occurs within a transitional context toward care models that, according to the Constitution and Comprehensive Health Care Model, aspire to transcend traditional biomedical paradigms by incorporating comprehensive, intercultural, and humanistic perspectives. However, effective implementation of these principles faces significant challenges from structural health system limitations, including insufficient resources, excessive workloads, and institutional cultures that prioritize technical efficiency over personalized care [17].

In this context, the training of nursing professionals presents specific factors that require greater attention. According to data from the Higher Education Quality Assurance Council [18], 21 universities offer nursing programs, 8 of which are public. By 2023, approximately 15,000 nursing students will be enrolled nationwide, with a graduation rate of 65%.

The higher education access system significantly shapes Ecuadorian students’ educational experiences. Some students enter nursing programs even though nursing was not their first choice, due to Higher Education Entrance Exam regulations [19]. This creates tension between students’ initial expectations about the program and the reality they encounter during their studies.

Training challenges at Ecuadorian public universities intensify with governmental changes. Budgetary constraints, insufficient infrastructure, and high student demand for nursing program admission create evident obstacles. The Secretariat of Higher Education, Science, Technology, and Innovation [20] reports that public university budgets have decreased by 12% over five years, affecting comprehensive training conditions and adequate nursing program capacity.

### 1.3. Research Gap and Study Rationale

Within this context, nursing students are uniquely positioned to experience care dehumanization tensions. As Fitzpatrick [21] in the United Kingdom, Mbalinda [22] in Africa, and Meléndez [23] in Mexico observe, students simultaneously inhabit educational and clinical contexts, experiencing through their own embodiment and subjectivity the contradictions between educational ideals and practical realities. From these experiences, they develop distinctive perspectives and creative strategies to preserve and revitalize care’s human dimensions, even within restrictive contexts.

In today’s healthcare environment, where global pandemics and health crises have intensified technical demands, the need for meaningful human care has become more pressing [24]. Paradoxically, while nursing has gained social recognition for its crucial role during these crises, the profession has also faced working conditions that challenge the fulfillment of its professional ideals [25,26,27]. Therefore, strengthening the profession’s humanistic values during academic training has become essential.

Based on these considerations, this study seeks to understand how nursing students experience, resist, and transform dehumanizing pressures from their environment, providing valuable insights not only for nursing education but also for guiding care practices that preserve humanistic values in increasingly technological and standardized healthcare systems. As Watson [28] argues, contemporary nursing faces the challenge of reaffirming its essence as human-centered care while integrating scientific and technological advances.

## 2. Materials and Methods

### 2.1. Study Design

We applied a phenomenological design based on the interpretive paradigm. This approach allowed us to understand “being” or “Dasein” in relation to care experiences. Heidegger [29] defines being as an existence that continuously relates to its environment and other beings. He emphasized that all descriptions represent interpretations of lived experience through a hermeneutic process grounded in four existential lifeworld concepts: spatiality, corporeality, temporality, and relationality [30,31]. Within this study’s framework, we explored how nursing students experience human care not as simple actions or techniques, but as fundamental aspects of their “being” and developing professional identity.

The methodology was developed through four systematic phases [32,33]. The first phase, called epoché, established an approach to the care phenomenon without prior ideas, theories, or hypotheses, favoring an open approach where meanings emerged directly from participants’ accounts. The second phase corresponded to phenomenological reduction, which focused on describing the phenomenon as it appears in students’ consciousness, focusing exclusively on direct experience of their pre-professional practices without adding interpretations or causal explanations. In the third phase, eidetic reduction was implemented, which through imaginative variation sought to capture the phenomenon’s essence, followed by transcendental reduction that examined the student’s consciousness role in care phenomenon apprehension. The process culminated in the fourth phase with phenomenological interpretation, where the phenomenon was rigorously detailed as captured in its essence by participants’ consciousness.

### 2.2. Study Setting

We conducted this research within a nursing program at a public higher education institution in northern Ecuador. This setting trains professionals with skills to provide comprehensive care to individuals, families, and communities. The mission and vision of this educational center emphasize training based on scientific, ethical, and humanistic principles to address the challenges of public and private healthcare.

The nine-semester program (four and a half years) includes eighth and ninth semester rotating internships in hospital and community units across Zone 1, which comprises Napo, Sucumbíos, and Imbabura provinces. During this period, students work shifts and comply with work schedules while receiving a government scholarship that covers housing and food expenses in their assigned province.

This educational context presents unique characteristics relevant to understanding student experiences. As a public institution serving diverse socioeconomic populations, students often balance academic demands with economic pressures, potentially influencing their perspectives on the tensions between efficiency and humanization. The rotating internship model, while providing diverse clinical exposures, also creates adaptation challenges that may intensify the technical–humanistic integration experiences explored in this study.

### 2.3. Participants

We purposively selected 17 students (12 women, 5 men) completing final-year pre-professional internships from 70 formally enrolled students. Participants were contacted by telephone from a list provided by the faculty supervising the rotating internship program. We used data saturation [34], reaching saturation after verifying that the final three interviews generated no new categories or relevant information beyond previously obtained data.

### 2.4. Data Collection

Data collection took place during the first quarter of 2025 through focused interviews using a semi-structured guide designed according to methodological considerations proposed by Bevan [35] and Guerrero [36]. The instrument included sociodemographic items and the following triggering question: “Can you describe your experience balancing technical demands with human aspects of care during your pre-professional internship?”. Supporting questions were asked when necessary to deepen understanding of participants’ experiences.

One researcher, a professor with master’s degree and qualitative research experience, conducted all interviews. Prior to data collection, the researcher established multiple friendly contacts with participants to build trust and create a comfortable interview environment. We audio-recorded interviews averaging 15–25 min per participant in school classrooms with only participant and interviewer present. We coded the 17 interviews using letter P and numerical order.

### 2.5. Data Analysis

Analysis followed a four-stage interpretive phenomenological approach [37]. We first read all transcripts multiple times to identify meaning units, then organized evidence according to the triggering question. Third, we established thematic meaning units and grouped them into subcategories. Finally, we developed main categories by identifying convergent patterns across all units.

Through sustained reflective reading of each transcript, 89 meaning units emerged and were consolidated into 18 subcategories, then reduced to six main categories. This manual analysis process prioritized understanding participants’ lived experiences rather than mechanical categorization, following van Manen’s [32] emphasis on reflective engagement with textual data.

Given nature of this study, the analysis focused on identifying meaningful patterns in students’ reported experiences rather than achieving the comprehensive hermeneutic interpretation typical of extended phenomenological studies. Each category preserved authentic student voices while revealing fundamental structures of their professional experiences.

### 2.6. Validation and Rigor

After interviews, we validated participants’ interpretations by having them review complete transcripts to confirm alignment with intended expressions, ensuring fidelity and credibility while recovering any missing meanings. We implemented research triangulation with three external analysts independently evaluating data and contrasting interpretations to reach consensus on final categories. No discrepancies emerged among reviewers during the expert triangulation process. Validations during data collection and analysis ensured credibility and transferability, maintaining methodological rigor [38]. We utilized the Consolidated Criteria for Reporting Qualitative Research (COREQ) checklist [39].

We acknowledge that the professor-student relationship between researcher and participants, while carefully managed through informed consent and anonymity assurances, may have influenced response patterns. To address this potential limitation, the validation process included explicit discussion of this dynamic during member checking, ensuring participants felt their authentic experiences were captured rather than idealized responses. The external analysts in the triangulation process were specifically briefed about this contextual factor when evaluating interpretive consistency.

### 2.7. Ethical Considerations

We met all ethical requirements, including obtaining informed consent from each participant and securing prior research protocol approval from the Universidad Técnica del Norte Faculty of Health Sciences under resolution UTN-CI-2024-269-R. We ensured voluntary participation, anonymity, no academic evaluation impact, confidential data handling, and clear procedures for audio recording storage and eventual destruction.

## 3. Results

Our sample consisted of 12 women (70.6%) and 5 men (29.4%), aged 22–31 years (mean age 24.5 ± 2.3 years). Seven participants (41.2%) completed practice rotations in community settings, while 10 (58.8%) completed rotations in hospital settings (Table 1). Community service students primarily described strategies for adapting to challenging socioeconomic conditions, while hospital rotation students emphasized time optimization strategies for care delivery.

Students in community settings (*n* = 7, 41.2%) faced resource limitations and socioeconomic barriers that shaped their humanization approaches. For example, P12 described adapting to material constraints: “Sometimes the health center doesn’t have gloves, so patients have to buy them, but many can’t afford to, so I give them some from my own supply.” This context fostered strategies centered on resourcefulness and direct personal support to maintain dignified care despite institutional limitations.

Students in hospital settings (*n* = 10, 58.8%) encountered high-volume, time-pressured environments that demanded efficiency-focused humanization strategies. P8 exemplified this challenge: “In the emergency department, it’s hard to find balance because there’s so much to do. You can’t just focus on one patient.” Hospital students developed skills in rapid therapeutic connection making, such as P6’s approach: “There’s always a moment during care to smile, make eye contact, talk, and offer a gentle touch,” integrating human contact within time constrained technical procedures.

The community context promoted sustained relationship building and holistic problem-solving, as students had more time per patient but fewer resources. Conversely, the hospital context required moments of humanization and efficient emotional connection, where students learned to maximize brief interactions’ therapeutic potential while managing multiple competing demands.

*Time Management and Optimization* reveals conscious strategies students developed to maximize technical and administrative efficiency, creating opportunities for greater attention to humanized care. This reflects complex organizational competency that reconciles healthcare system productivity demands with professional humanistic values (Table 2).

*Integration of Human and Technical Dimensions* describes deliberate actions students developed to simultaneously merge technical care aspects with elements that enable humanized care provision. Students transformed routine technical moments into therapeutic connection opportunities (Table 2).

*Patient Communication About Time Constraints* describes specific communication skills students developed to manage patient expectations during time limitations, care overload, or circumstances that prevent immediate attention. This reflects specialized communication skills that maintain therapeutic relationships despite adverse conditions (Table 2).

*Emotional Regulation and Boundary Setting* encompasses how students manage emotional responses to caregiving situations while establishing healthy personal-professional boundaries, balancing empathic involvement necessary for human care with emotional well-being protection (Table 2).

*Resistance to Dehumanization* encompasses conscious strategies students developed to preserve and defend humanistic care aspects against institutional, cultural, and systemic pressures that reduce nursing practice to purely technical components.

*Institutional Context Adaptation* encompasses strategies students developed to adjust professional practice to institutional environment demands, limitations, and characteristics during internships, involving constant negotiation between humanized care ideals and actual health system constraints (Table 2).

## 4. Discussion

Student narratives reveal existential tension between technical skill demands and humanized care imperatives, creating experiences where students apply creative integration and resistance strategies.

*Time Management and Optimization* relates to Heidegger’s fundamental ontological definition of Dasein as a specific mode of human being [29]. Students experience the ability to transform institutional chronological time (chronos) into meaningful lived time (kairos) during authentic care encounters. Through direct clinical situations, students’ preconceived understanding shifts as organizational time transforms into more complex dimensions, including time management that prioritizes authentic therapeutic presence with patients.

These experiences reveal that students conceive care not solely from efficiency perspectives but as requiring genuine therapeutic encounters with care recipients. These insights align with studies showing student progression from technical and professional focus in early academic years toward psychosocial factors in later years [40,41,42].

*Integration of Human and Technical Dimensions* reveals deep understanding of care embodiment. Real situation encounters constitute integral care acts where caregiver and patient bodies meet in shared vulnerability and mutual empowerment. These findings align with Ponty’s embodiment concepts, cited by González [43], where the body represents an attitude relating to present tasks, with space serving as the medium for this possibility.

Students experienced care corporeally such that each physical contact during technical care moments became opportunities for authentic human encounters. Through this dimension, students developed comprehensive care understanding where each gesture during performed procedures became therapeutic patient connection opportunities, supporting Guerrero’s [12] argument that caring moments transcend time, space, and embodiment.

*Patient Communication About Time Constraints* demonstrates specific communication skill development toward expectation management and conflict prevention. This direct encounter with situations requiring time constraint communication reveals care’s ethical dimension, demonstrating student responsibility toward others that transcends care’s ethical essence.

Communicative competence serves as a strategy for managing patient expectations during care overload stress. Testimonies demonstrate how students develop sensitivity in word selection that maintains therapeutic relationships despite pressure, consistent with research by Rosas [44] and Gregory [45] emphasizing communication skill needs for comprehensive care.

*Emotional Regulation and Boundary Setting* describes processes through which students manage emotional responses to caregiving situations while establishing healthy boundaries between empathic involvement and personal well-being preservation. Student narratives reveal the perceived complexity of achieving emotional regulation, seeking not to eliminate emotional involvement but to modulate it to preserve both care quality and personal well-being.

This regulation involves dynamic balance between controlled vulnerability and self-protection, as Restrepo [46] asserts. Navarro et al. [47] argue that appropriate student emotion management relates to physical and psychological well-being, personal satisfaction, and improved academic performance, highlighting the need for curriculum changes that integrate communication and emotional skill development.

*Resistance to Dehumanization* represents significant findings that demonstrate student ability to preserve and defend nursing’s humanistic values against institutional and cultural pressures. This ontological resistance constitutes authentic nursing nature preservation against healthcare system dehumanizing influences, finding theoretical foundation in Díaz et al. [48] analysis arguing that many healthcare institutions maintain biomedical model approaches that focus on procedures rather than person-centered integrity.

This resilience manifested in multiple ways: from conscious decisions to maintain meaningful patient interactions despite supervisor criticism to explicit refusal to adopt perceived dehumanizing professional models. Students developed resilient professional identity that navigates tension between efficiency demands and humanized care professional values, relating to Watson’s Care Theory [28,49,50] on preserving care essence against practice technologization and bureaucratization.

The sixth category encompasses the strategies students develop to adapt their professional practice to environmental limitations. This involved a constant negotiation process between humanized care ideals and actual health system constraints. However, while Reyes et al. [1] describe these barriers as obstacles for professionals who have completed their training, the present study provides a novel perspective on how students in training develop strategies during their pre-professional practices, transforming system restrictions into adaptive learning opportunities.

### 4.1. Curricular Implications

These findings suggest nursing education must develop specific competencies for preserving humanistic values alongside technical training. Core curriculum should include therapeutic communication under pressure, emotional regulation and boundary-setting, adaptive time management that creates space for human connection, and strategies for resisting institutional dehumanization while maintaining professional effectiveness.

Training programs should use realistic simulations that reproduce technical-humanistic tensions before clinical rotations. Clinical supervision must integrate technical and interpersonal skill development, providing feedback on both procedural competence and therapeutic relationship maintenance within time-pressured environments.

This integrated approach prepares students to transform institutional limitations into opportunities for compassionate, efficient care rather than viewing humanity and efficiency as competing priorities.

### 4.2. Limitations

Study limitations include focus on the pre-professional internship phase, not capturing initial or final semester student experiences that could reveal integrative competency development differences during academic training. Interview duration was relatively short for phenomenological studies, potentially limiting narrative depth. This brevity resulted not only from organizational constraints but also from participants’ concise responses. Despite interviewer efforts to elicit richer narratives, some participants proved less communicative during interviews, providing limited elaboration on their experiences.

Additionally, findings reflect Ecuadorian socioeconomic and cultural realities, requiring further Latin American and international context studies to identify universal patterns and specificities in technical-humanistic integration strategy development. Sample selection was limited to a single university and restricted geographical area, reducing finding transferability.

## 5. Conclusions

Nursing students develop six complementary strategies to integrate technical efficiency with humanized care: time optimization, procedural-relational integration, patient communication about constraints, emotional regulation, resistance to dehumanization, and institutional adaptation. These strategies demonstrate that efficiency and humanization are not opposing forces but can be synergistic when skillfully managed.

Students actively transform technical procedures into therapeutic opportunities while resisting institutional pressures that reduce nursing to purely technical practice. They develop specialized communication skills for resource-limited environments and maintain emotional boundaries that protect both care quality and personal well-being.

Nursing education must incorporate competencies that preserve humanistic values alongside technical training: adaptive time management, therapeutic communication under pressure, emotional regulation, and resistance strategies against dehumanizing forces. Clinical supervision should emphasize integrated technical-interpersonal development rather than treating these as separate domains.

This study reveals how nursing students preserve professional humanistic values within technological healthcare systems, transforming systemic limitations into opportunities for resilient, integrated practice that honors both scientific excellence and compassionate care.

## Figures and Tables

**Table 1:** Characterization of the participants.

Student	Semester	Age	Sex	Clinical Context
P1	Ninth	31	Female	Hospital
P2	Eighth	23	Female	Hospital
P3	Eighth	24	Female	Hospital
P4	Eighth	24	Male	Hospital
P5	Eighth	22	Female	Hospital
P6	Eighth	24	Female	Hospital
P7	Eighth	28	Female	Hospital
P8	Eighth	22	Female	Community
P9	Ninth	23	Male	Hospital
P10	Eighth	23	Female	Hospital
P11	Eighth	23	Female	Hospital
P12	Ninth	24	Male	Community
P13	Ninth	26	Female	Community
P14	Ninth	25	Male	Community
P15	Ninth	26	Male	Community
P16	Eighth	26	Female	Community
P17	Ninth	24	Female	Community

**Table 2:** Categories and subcategories that emerged from the nursing students’ illustrative quotes.

Categories	Subcategories	Illustrative Quotes
*Time Management and Optimization*	Task organization; Technical efficiency; Documentation management.	“First, I get organized. I prepare medications for scheduled times and perform procedures like IV line placement and wound care.” (P3); “To achieve balance, we need to be skilled at procedures so we don’t delay care and can optimize our time.” (P6); “I need to complete records and reports faster so I can spend more time with patients.” (P17)
*Integration of Human and Technical Dimensions*	Communication during procedures; Holistic assessment; Interaction maximization	“While I am giving medication, I explain everything to the patient and ask how they’re feeling at the same time.” (P4); “While I assess them, I greet them warmly, chat with them, and evaluate their condition before documenting my findings.” (P3); “There’s always a moment during care to smile, make eye contact, talk, and offer a gentle touch.” (P6); “When I perform a procedure, I always like to talk with patients and explain everything I’m going to do.” (P12)
*Patient Communication About Time Constraints*	Proactive explanation; Carefully chosen language; Transparency about limitations	“I explain to the patient and ask them to please wait a moment because we’re swamped.” (P14); “I always choose my words carefully to explain that care might be delayed by five or ten minutes due to situations I can’t control.” (P15); “When we have many patients, I first explain the situation and ask them to wait, being kind and friendly so they understand instead of getting upset with me.” (P11); “I try to politely explain why their care is being delayed.” (P16).
*Emotional Regulation and Boundary Setting*	Emotional control; Boundary establishment; Priority discernment; Personal limitations	“My approach is to stay focused, keep calm, and try to understand why people react the way they do.” (P16); “I’ve learned that I need to be sensitive to patients’ situations as much as I can handle.” (P2); “Sometimes I go home after my shift, and I’m still worried about my patients.” (P1); “Finding balance is hard because I’m a very emotional and sensitive person.” (P11).
*Resistance to dehumanization*	Professional identity construction; Consistent compassion; Deliberate humanization; Negative role model identification	“I don’t want to become a cold, technical professional. I want to keep focusing on the human side of our profession, which many people are losing because of all the technology and routine.” (P9); “At the hospital, they’re always telling me not to get too attached to patients, not to talk so much because I have other things to do.” (P17); “I’ve watched professionals who just focus on the technical aspects of care without any emotional connection. I worry about becoming like that, so I try not to follow their example.” (P1); “Nurses often scold me for talking and connecting with patients. They’ve even told my supervisor that I’m too slow, but I just can’t help myself, I can’t stop doing it.” (P11)
*Institutional Context Adaptation*	Service-specific strategies; Workload management; Institutional expectation negotiation; Resource	“ In the emergency department, it’s hard to find balance because there’s so much to do. You can’t just focus on one patient.” (P8); “When we have many patients and one gets complicated, it’s difficult to maintain balance between technical and human care for everyone.” (P7); “Sometimes the health center doesn’t have gloves, so patients have to buy them, but many can’t afford to, so I give them some from my own supply.” (P12); “The workload is really heavy, and it was hard to adapt at first. Sometimes I want to provide both technical and human care with more quality time for my patients, but it’s complicated. That’s what my supervisors notice most about me.” (P10)

## Data Availability

Data supporting study conclusions are available from the corresponding author upon reasonable request, subject to privacy policy compliance and participant anonymization requirements.
